# Supplementary material for: From sequence to enzyme mechanism using multi-label machine learning
Source: BMC Bioinformatics. 2014 May 19;15:150. doi: 10.1186/1471-2105-15-150 (PMC4229970; doi:10.1186/1471-2105-15-150)
Supplement: Additional file 2 — Java code of ml2db. Additional file ml2db_code.tar.gz contains the Java source code to run the multi-label machine learning experiments and save the results to database. The code’s Javadoc is included. [file 1471-2105-15-150-S2.zip › additional file 2/ml2db/ml2db/doc/main/ml2db/al/learning/AlExperimenter.html]

AlExperimenter


JavaScript is disabled on your browser.


- Overview
- Package
- Class
- Use
- Tree
- Deprecated
- Index
- Help

- Prev Class
- Next Class

- Frames
- No Frames

- All Classes

- Summary:
- Nested |
- Field |
- Constr |
- Method

- Detail:
- Field |
- Constr |
- Method


main.ml2db.al.learning

## Class AlExperimenter

- java.lang.Object
- - main.ml2db.mulan.learn.crosseval.CrossExperimenter
  - - main.ml2db.mulan.learn.traintest.TrainTestExperimenter
    - - main.ml2db.al.learning.AlExperimenter

- ---

    

  ```
  public class AlExperimenter
  extends TrainTestExperimenter
  ```

  Class

  Version:
  :   14 Jul 2011

  Author:
  :   Luna De Ferrari ldf(at)st-andrews.ac.uk

- - ### Constructor Summary

    Constructors

    Constructor and Description

    `AlExperimenter(mulan.data.MultiLabelInstances trainSet,
    mulan.data.MultiLabelInstances testSet,
    DbManagers dbMan,
    MulanLearner learner,
    java.lang.String resultsDirectory,
    ActiveLearning experiment)`
  - ### Method Summary

    Methods

    | Modifier and Type | Method and Description |
    | --- | --- |
    | `void` | `evaluateAndSave()` |
    | `ActiveLearning` | `getActiveLearner()` |
    | `AlOutputManager` | `getOutputManager()` |
    | `void` | `setOutputManager(uk.ac.standrews.utils.main.database.DbManager resultsDbManager, java.lang.String resultsDirectory, uk.ac.standrews.utils.main.database.Table table)` |

    - ### Methods inherited from class main.ml2db.mulan.learn.traintest.TrainTestExperimenter

      `evaluateAndSave, getEvaluator, getInputManager, getNewEvaluator, main, run, setInputManager, setInputManager, setInputManager`
    - ### Methods inherited from class main.ml2db.mulan.learn.crosseval.CrossExperimenter

      `evaluateAndSave, getLearner, getTestArffId, getTrainArffId, run, setTestArffId`
    - ### Methods inherited from class java.lang.Object

      `equals, getClass, hashCode, notify, notifyAll, toString, wait, wait, wait`

- - ### Constructor Detail


    - #### AlExperimenter

      ```
      public AlExperimenter(mulan.data.MultiLabelInstances trainSet,
                    mulan.data.MultiLabelInstances testSet,
                    DbManagers dbMan,
                    MulanLearner learner,
                    java.lang.String resultsDirectory,
                    ActiveLearning experiment)
      ```
  - ### Method Detail


    - #### evaluateAndSave

      ```
      public void evaluateAndSave()
      ```


    - #### getActiveLearner

      ```
      public ActiveLearning getActiveLearner()
      ```


    - #### getOutputManager

      ```
      public AlOutputManager getOutputManager()
      ```

      **Overrides:**
      :   `getOutputManager` in class `TrainTestExperimenter`


    - #### setOutputManager

      ```
      public void setOutputManager(uk.ac.standrews.utils.main.database.DbManager resultsDbManager,
                          java.lang.String resultsDirectory,
                          uk.ac.standrews.utils.main.database.Table table)
      ```

      **Overrides:**
      :   `setOutputManager` in class `TrainTestExperimenter`


- Overview
- Package
- Class
- Use
- Tree
- Deprecated
- Index
- Help

- Prev Class
- Next Class

- Frames
- No Frames

- All Classes

- Summary:
- Nested |
- Field |
- Constr |
- Method

- Detail:
- Field |
- Constr |
- Method
